# Lessons learned after one year of COVID-19 from a urologist and radiotherapist view: A German survey on prostate cancer diagnosis and treatment

**DOI:** 10.1371/journal.pone.0269827

**Published:** 2022-06-14

**Authors:** Nina N. Harke, Christian Wagner, Robert M. Hermann, Boris A. Hadaschik, Jan Philipp Radtke, Alev Altay-Langguth, Stefan Aufderklamm, Christian Bach, Martina Becker-Schiebe, Andreas Blana, Frank Bruns, Stephan Buse, Stephanie E. Combs, Christina L. Engels, Emad Ezzibdeh, Marcel Fiedler, Laura-Anna Fischer, Mahmoud Farzat, Alexander Frismann, Matthias M. Heck, Christoph Henkenberens, Marie C. Roesch, Christoph Käding, Gunther Klautke, Philipp Krausewitz, Markus A. Kuczyk, Conrad Leitsmann, Sebastian Lettmaier, Samy Mahjoub, Andreas Manseck, Daniel Medenwald, Andreas Meyer, Oliver Micke, Rudolf Moritz, Marcel Ott, Inga Peters, Sasa Pokupic, Daniel Porres, Felix Preisser, Kathrin Reichel, Andreas Schneider, Christian Schwentner, Sergiu Scobioala, Michael Truss, Daniel Wegener, Felix Wezel, Kay Willborn, Jörn H. Witt, Andrea Wittig, Michael Wittlinger, Hendrik A. Wolff, Volker Zimmermanns, Hans Christiansen

**Affiliations:** 1 Department of Urology, Hannover Medical School, Hannover, Germany; 2 Department of Urology, St. Antonius Hospital, Gronau, Germany; 3 Center of Radiotherapy, Bremen, Germany; 4 Department of Urology, University of Duisburg-Essen, Essen, Germany; 5 Department of Radiotherapy, University Hospital, Goethe University, Frankfurt am Main, Germany; 6 Department of Urology, Eberhard Karls University Tuebingen, Tuebingen, Germany; 7 Department of Urology, RWTH Aachen University, Aachen, Germany; 8 Department of Radiotherapy, Klinikum Braunschweig, Braunschweig, Germany; 9 Department of Urology, Fürth Hospital, Fürth, Germany; 10 Department of Radiotherapy, Hannover Medical School, Hannover, Germany; 11 Department of Urology, Alfried Krupp Krankenhaus, Essen, Germany; 12 Department of Radiation Oncology, Technical University of Munich, Munich, Germany; 13 Institute of Radiation Medicine, Helmholtz Zentrum München (HMGU), Oberschleißheim, München, Germany; 14 Deutsches Konsortium für Translationale Krebsforschung (DKTK), Partner Site Munich, Munich, Germany; 15 Department of Urology, Municipal Hospital Karlsruhe, Karlsruhe, Germany; 16 Department of Urology, Klinikum Lüneburg, Lueneburg, Germany; 17 Department of Urology, SLK Kliniken Heilbronn, Heilbronn, Germany; 18 Department of Radiotherapy, University Medical Center Goettingen, Goettingen, Germany; 19 Department of Urology, Diakonie Klinikum, Siegen, Germany; 20 Department of Radiotherapy, University of Leipzig, Leipzig, Germany; 21 Department of Urology, Technical University of Munich, Munich, Germany; 22 Department of Radiotherapy, University Hospital Bonn, Bonn, Germany; 23 Department of Urology, University Hospital Schleswig-Holstein, Luebeck, Germany; 24 Department of Urology, University of Medicine Greifswald, Greifswald, Germany; 25 Clinic for Radiation Oncology, Chemnitz Medical Center, Chemnitz, Germany; 26 Department of Urology, University Hospital Bonn, Bonn, Germany; 27 Department of Urology, University Medical Center Goettingen, Goettingen, Germany; 28 Department of Radiation Oncology, Universitätsklinikum Erlangen, Erlangen, Germany; 29 Department of Urology, Klinikum Ingolstadt, Ingolstadt, Germany; 30 Department of Radiation Therapy, University Hospital Halle/Saale, Halle, Germany; 31 Center of Radiotherapy Hildesheim/Goslar, Hildesheim, Germany; 32 Department of Radiotherapy, Franziskus Hospital Bielefeld, Bielefeld, Germany; 33 Department of Urology, Marien Hospital, Ruhr-University Bochum, Herne, Germany; 34 Center of Radiotherapy Wolfsburg, Wolfsburg, Germany; 35 Department of Urology, Asklepios Klinikum Harburg, Hamburg, Germany; 36 Department of Urology, Klinikum Leverkusen, Leverkusen, Germany; 37 Department of Urology, University Hospital, Goethe University, Frankfurt am Main, Germany; 38 Department of Urology, Sana Klinikum Hof, Hof, Germany; 39 Department of Urology, Main-Kinzig-Kliniken Standort Gelnhausen, Gelnhausen, Germany; 40 Department of Urology, Diakonieklinikum Stuttgart, Stuttgart, Germany; 41 Department of Radiation Oncology, University Hospital Muenster, Muenster, Germany; 42 Department of Urology, Klinikum Dortmund, Dortmund, Germany; 43 Department of Radiation Oncology, University of Tübingen, Tuebingen, Germany; 44 Department of Urology, Ulm University Hospital, Ulm, Germany; 45 Department of Radiotherapy, Pius Hospital Oldenburg, Oldenburg, Germany; 46 Department of Radiotherapy and Radiation Oncology, University of Jena, Jena, Germany; 47 Department of Radiotherapy, Fürth Hospital, Fuerth, Germany; 48 Department of Radiology, Nuclear Medicine and Radiotherapy, Radiologie München, Munich, Germany; 49 Department of Urology, Siloah St. Trudpert Klinikum, Pforzheim, Germany; Carolina Urologic Research Center, UNITED STATES

## Abstract

**Introduction:**

Since the beginning of the pandemic in 2020, COVID-19 has changed the medical landscape. International recommendations for localized prostate cancer (PCa) include deferred treatment and adjusted therapeutic routines.

**Materials and methods:**

To longitudinally evaluate changes in PCa treatment strategies in urological and radiotherapy departments in Germany, a link to a survey was sent to 134 institutions covering two representative baseline weeks prior to the pandemic and 13 weeks from March 2020 to February 2021. The questionnaire captured the numbers of radical prostatectomies, prostate biopsies and case numbers for conventional and hypofractionation radiotherapy. The results were evaluated using descriptive analyses.

**Results:**

A total of 35% of the questionnaires were completed. PCa therapy increased by 6% in 2020 compared to 2019. At baseline, a total of 69 radiotherapy series and 164 radical prostatectomies (RPs) were documented. The decrease to 60% during the first wave of COVID-19 particularly affected low-risk PCa. The recovery throughout the summer months was followed by a renewed reduction to 58% at the end of 2020. After a gradual decline to 61% until July 2020, the number of prostate biopsies remained stable (89% to 98%) during the second wave. The use of RP fluctuated after an initial decrease without apparent prioritization of risk groups. Conventional fractionation was used in 66% of patients, followed by moderate hypofractionation (30%) and ultrahypofractionation (4%). One limitation was a potential selection bias of the selected weeks and the low response rate.

**Conclusion:**

While the diagnosis and therapy of PCa were affected in both waves of the pandemic, the interim increase between the peaks led to a higher total number of patients in 2020 than in 2019. Recommendations regarding prioritization and fractionation routines were implemented heterogeneously, leaving unexplored potential for future pandemic challenges.

## Introduction

When the coronavirus disease 2019 (COVID-19) pandemic started, the explosive increase in infections and deaths in the first weeks of February and March 2020 resulted in unprecedented changes in health care systems worldwide. Multiple recommendations were developed to meet these challenges in urology [1] as well as radiation oncology [2]. These suggestions, including triage systems and deferral policies, affected cancer patients in terms of delayed diagnosis, consultation visits and postponements of therapies.

Due to the heterogeneity of the disease, prostate cancer (PCa) patients choose between various treatment options depending on their individual risk group [3, 4]. While active surveillance is a valid alternative for low-risk cases with an excellent long-term outcome [5, 6], the oncological consequences of deferred treatment of unfavorable PCa remain inconclusive [7]. The COVID-19 outbreak delayed therapy even for patients with the highest risk in a variety of hospitals. According to the European Association of Urology (EAU), surgical treatment with radical prostatectomy (RP) could be postponed for at least three months or until after the pandemic, and external beam radiation therapy (EBRT) was considered a guideline-compliant alternative [8]. While deferral was the primary recommendation for radiation therapy for low-risk patients, various working groups pointed out that alternative shorter treatment regimens are available; thus, with reduced fractions in radiotherapy series, the risk of COVID-19 exposition and transmission during the reduced visits in hypofractionation can be anticipated [9–11].

This study aims to provide insight into the situation and changes in diagnostic and therapeutic strategies for localized PCa from the urological and radiation oncology perspective since the COVID-19 outbreak in Germany.

## Material and methods

### Online survey

Based on a previous study initiated by the “Laparoscopy and robot-assisted surgery” working group of the German Association of Urology [12], an online questionnaire with a focus on PCa was conceptualized using the Google Docs open-source survey tool [13]. The survey was conducted in accordance with the Checklist of Reporting Results of Internet E-surveys (CHERRIES) [14]. On March 9, 2021, the heads of 84 German urological departments and 50 institutions specializing in radiotherapy for PCa were contacted via email that included a link to the online survey. Data collection was closed on May 30, 2021.

The first section of the survey included baseline information regarding the institution (city, state, type: academic/nonacademic, public/private) and the case numbers of patients with primary local treatment for localized prostate cancer (radiotherapy or radical prostatectomy) in 2019 and 2020. The second section comprised 15 subsections with repeated questions for the following timepoints: Baseline week 1 and baseline week 2, which represented two regular weeks before the pandemic, March 16 to March 22, 2020; April 20 to April 26, 2020; May 18 to May 24, 2020; June 15 to June 21, 2020; July 13 to July 19, 2020; Aug 10 to Aug 16, 2020; Sept 7 to Sept 13, 2020; Oct 5 to Oct 11, 2020; Nov 2 to Nov 8, 2020; Nov 30 to Dec 6, 2020; Dec 14 to Dec 20, 2020; Jan 11 to Jan 17, 2021; and Feb 8 to Feb 14, 2021. Each week, members of both specialties were asked about the numbers of localized prostate cancer patients undergoing either radical prostatectomy or their first course of radiotherapy with subcategorization in low-, intermediate- and high-risk groups according to the D’Amico classification [4]. Further questions for radiotherapists included case numbers for conventional and hypofractionation regimens. In addition, the participating urologists provided numbers of prostate biopsies (PBx) and radical prostatectomies.

In consultation with the local ethics committee, IRB approval and patient informed consent were not necessary for this study due to the lack of patient-related outcomes. The survey respondents gave written consent to study participation at the end of the questionnaire.

### Daily situation report—Robert Koch Institute

The German situation report of the pandemic is updated daily on the homepage of the German Government’s agency for disease control and prevention, “Robert Koch Institute” (RKI) [15].

### Statistical analyses

SPSS 25 was used for statistical analysis. Baseline week represents the mean of baseline week 1 and baseline week 2. Categorical data are shown as frequencies and proportions; continuous variables are given assums (total numbers) or medians with ranges. The chi-square test was used for categorical data with a statistical significance of p<0.05.

## Results

Forty-seven out of 134 questionnaires (35%) were completed by 27 urological and 20 radiation oncology departments. The majority of responses were collected from university hospitals (47%), followed by academic teaching hospitals (32%), nonacademic institutions (13%) and four private practices for radiation oncology (8%). Seventy-nine percent of the replies were from public hospitals, while 21% of the replies were from private institutions.

### Development of prostate cancer case numbers

**Fig 1:**
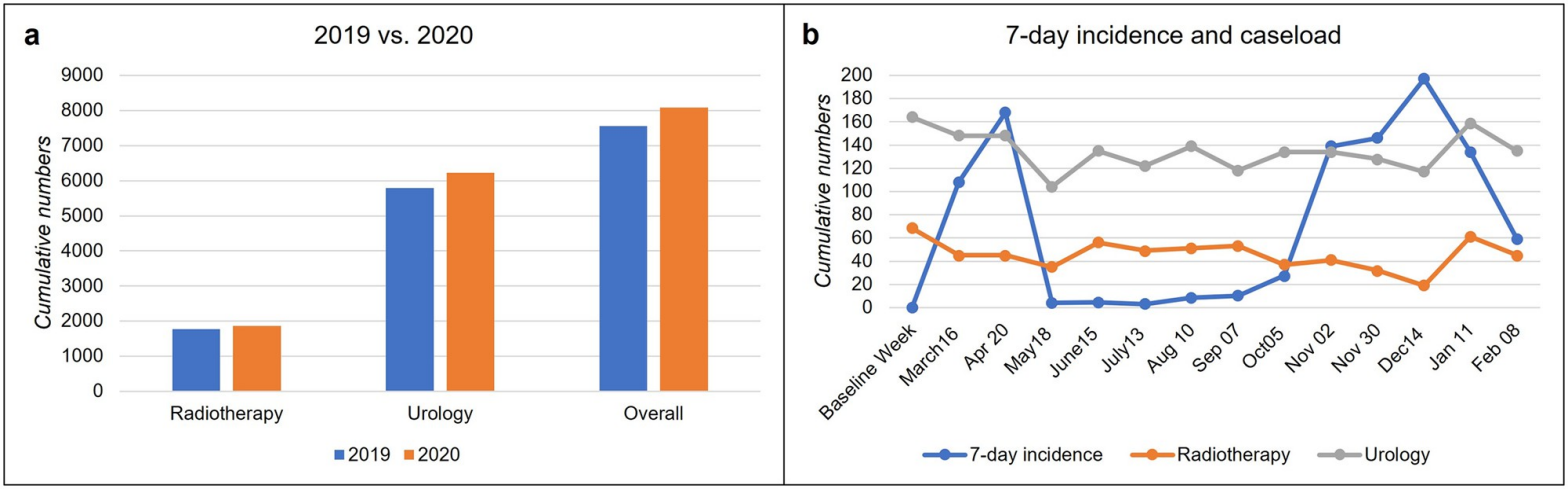
Cumulative numbers of PCa patients. (A) Cumulative numbers of patients undergoing prostate cancer therapy either with radiotherapy or radical prostatectomy in 2019 vs. 2020. (B) Development of patient numbers after the beginning of the pandemic. The blue line demonstrates the 7-day incidence of COVID-19 in the corresponding week.

Compared to 2019, we found an overall increase in local therapies for PCa by 6% (7560 vs. 8085 in 2020) in the participating institutions. Radiation therapy cases increased by 5% (1770 vs. 1858), and RPs increased by 7% (5790 in 2019 vs. 6227) (Fig 1A). A total of 69 EBRT procedures (median 2.75 EBRT/center) and 164 RPs occurred (median 4.5 RP/center) in the representative baseline week before the COVID-19 pandemic was documented. In the following weeks of the first wave of COVID-19, the overall numbers decreased to 60% with a maximum decline in May 2020, with reductions in EBRT down to 51% and RPs to 63%. The second wave (beginning mid-November 2020) showed a similar impact with an overall drop to 58%: the patient numbers decreased to 28% and 60% for EBRT and 71% to 82% for RPs of the initial total caseload. While the total numbers recovered in the summer months between the first and second waves, the initial caseload numbers could not be reached in either specialty except for January 2021 (Fig 1B).

The previously mentioned decrease included particularly low-risk PCa patients during the first wave, dropping to 41% in May 2020. This rate persisted at a low level until fall 2020. The treatment of intermediate- and high-risk PCa patients remained stable, with 91 to 97%, respectively. until April 2020, with a decline in the following months and fluctuations during the summer. Preceding the second wave, the therapy of low-risk PCa exceeded the baseline total patient number by 14%, mainly because of an increase in RPs in October and November 2020. In contrast, we found a distinct decrease in the cumulative numbers of high-risk patients in November and December, primarily in radiation therapy (down to 30% in December) but also in surgical treatment, with a decline to 64%.

### Prostate biopsy and radical prostatectomy

**Fig 2:**
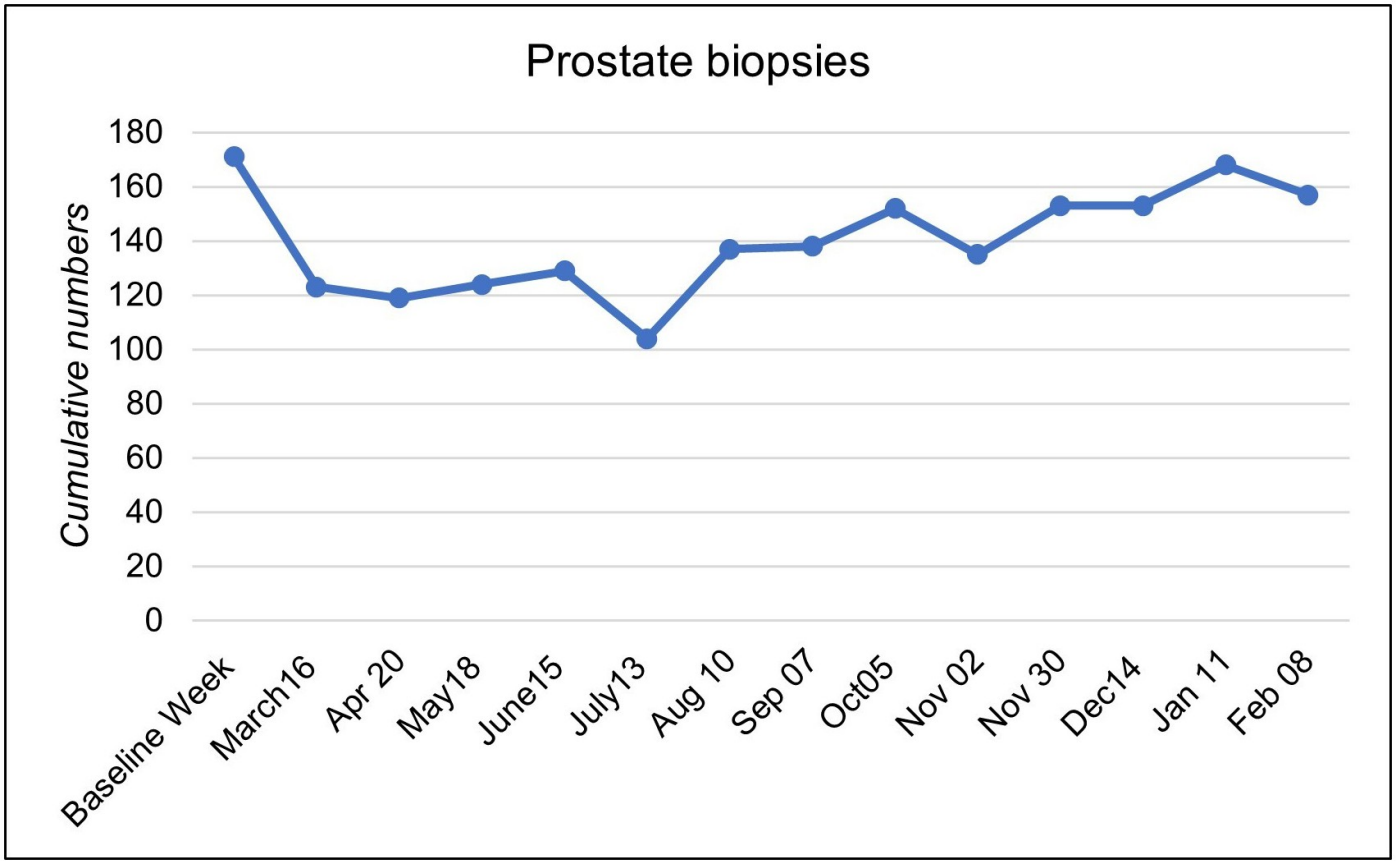
Changes in prostate biopsies. Total numbers of prostate biopsies performed in the baseline week and after the onset of the pandemic.

Following the overall case development, the numbers of PBx decreased after the declaration of the pandemic. The respondents reported a gradual decline from 171 biopsies in the baseline week to 123 in March 2020 (72%) to a minimum of 104 in July (61%). Then, a rise in PBx to 80% of the baseline numbers was observed. Following a fluctuation period in the fall, a further increase from 89% up to 98% was documented after November 30, 2020, with stable numbers during the second wave (Fig 2).

**Fig 3:**
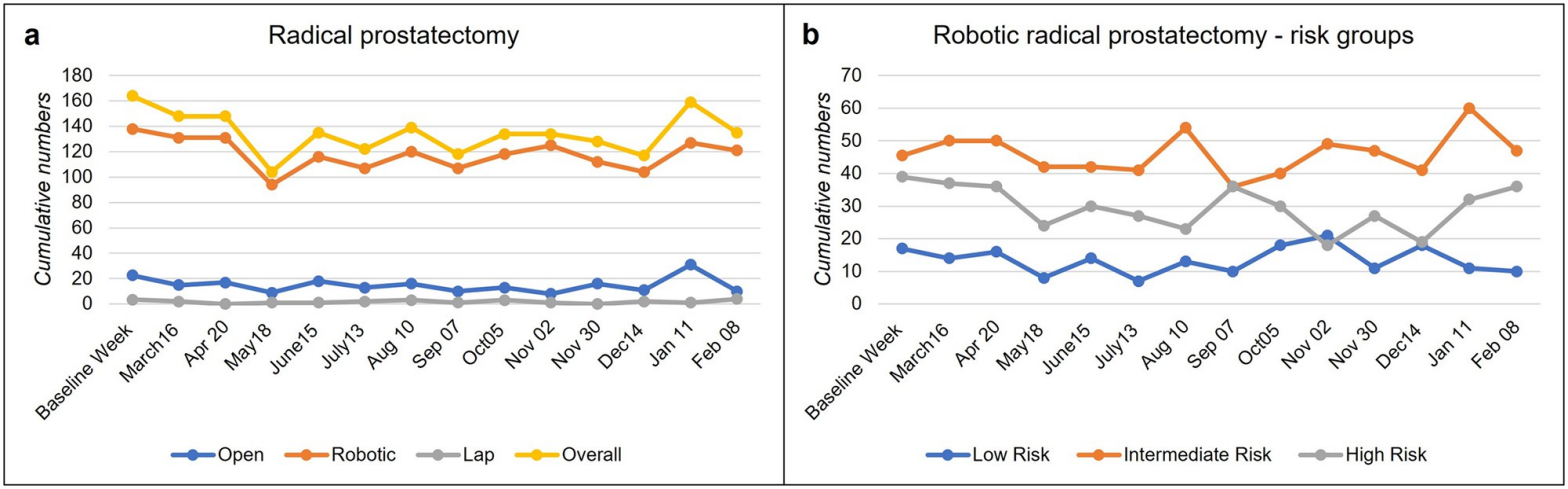
Changes in radical prostatectomies. (A) Overall numbers of radical prostatectomies classified as open, laparoscopic and robotic approaches and risk groups according to the D’Amico score (B).

RPs were exclusively performed robotically in 16 institutions (median 4.75 RP/center in the baseline week (range: 1–31)), in one laparoscopically, one center offered a laparoscopic and open approach, and nine hospitals performed both open and robotic RPs (median 3 RP/center in the baseline week (range 2–15)). None of the minimally invasive operation centers changed their routine to open prostatectomy. In those ten institutions offering both techniques, the proportion of the open approach remained stable with a baseline of 38% compared to 62% robotic prostatectomy (Fig 3A). Centers that only offered robotic RP documented no changes in March and April and a decrease to 74% in May as well as 78% in December. Robotic surgery for high-risk PCa fluctuated during the pandemic (46 to 92%) and never exceeded the baseline numbers, while cases with a low and intermediate risk periodically surpassed the 100% threshold (low-risk 41 to 124%, intermediate-risk 79 to 132% of the initial total caseload) (Fig 3B).

### Radiation therapy

**Fig 4:**
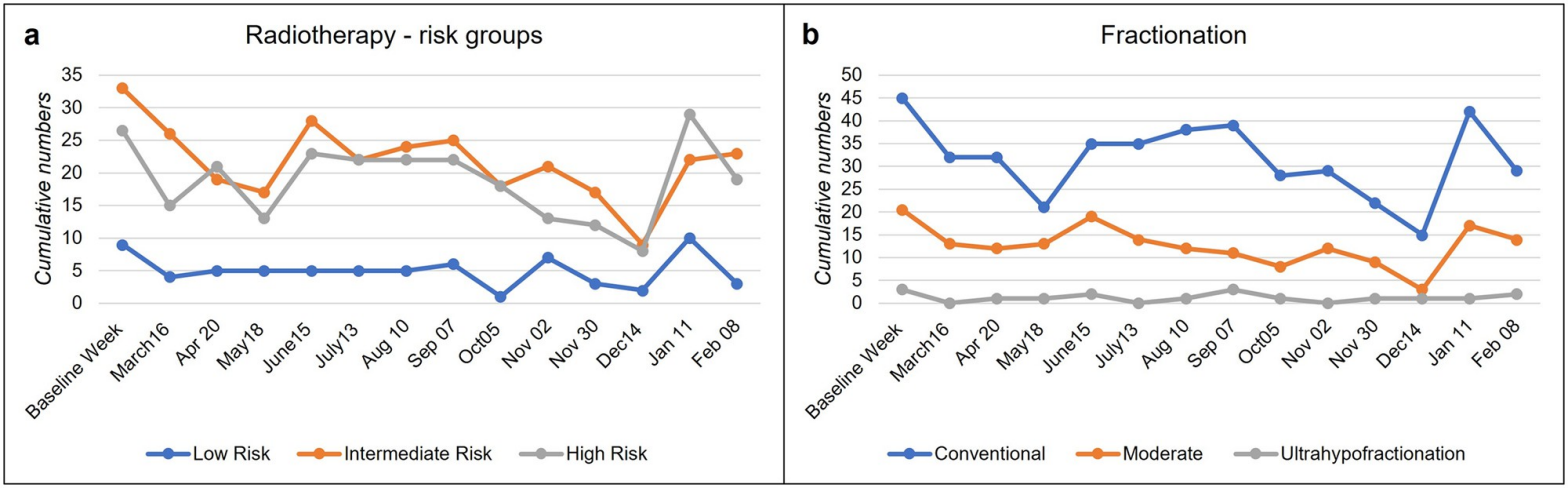
Changes in radiotherapy. (A) Cumulative numbers of patients treated with radiotherapy with division in PCa risk groups and type of fractionation (B).

Radiation therapy was deferred for the majority of low-risk patients during the first and second waves. While the first weeks of the pandemic had a similar impact on the intermediate- and high-risk groups, with a maximum decrease in May 2020 (52% and 49%), the numbers recovered, especially for high-risk PCa, to a range of 83% to 87% between June and September, followed by an additional decline during the second wave (Fig 4A).

In the baseline week before the pandemic, 50% of the respondents reported the use of hypofractionation, with no statistically significant difference between private and public institutions (both 50%, p = 1.00). Conventional fractionation was the standard of care in most centers, with 66% of the EBRT cases followed by moderate hypofractionation (30%) and ultrahypofractionation (4%) (Fig 4B).

**Fig 5:**
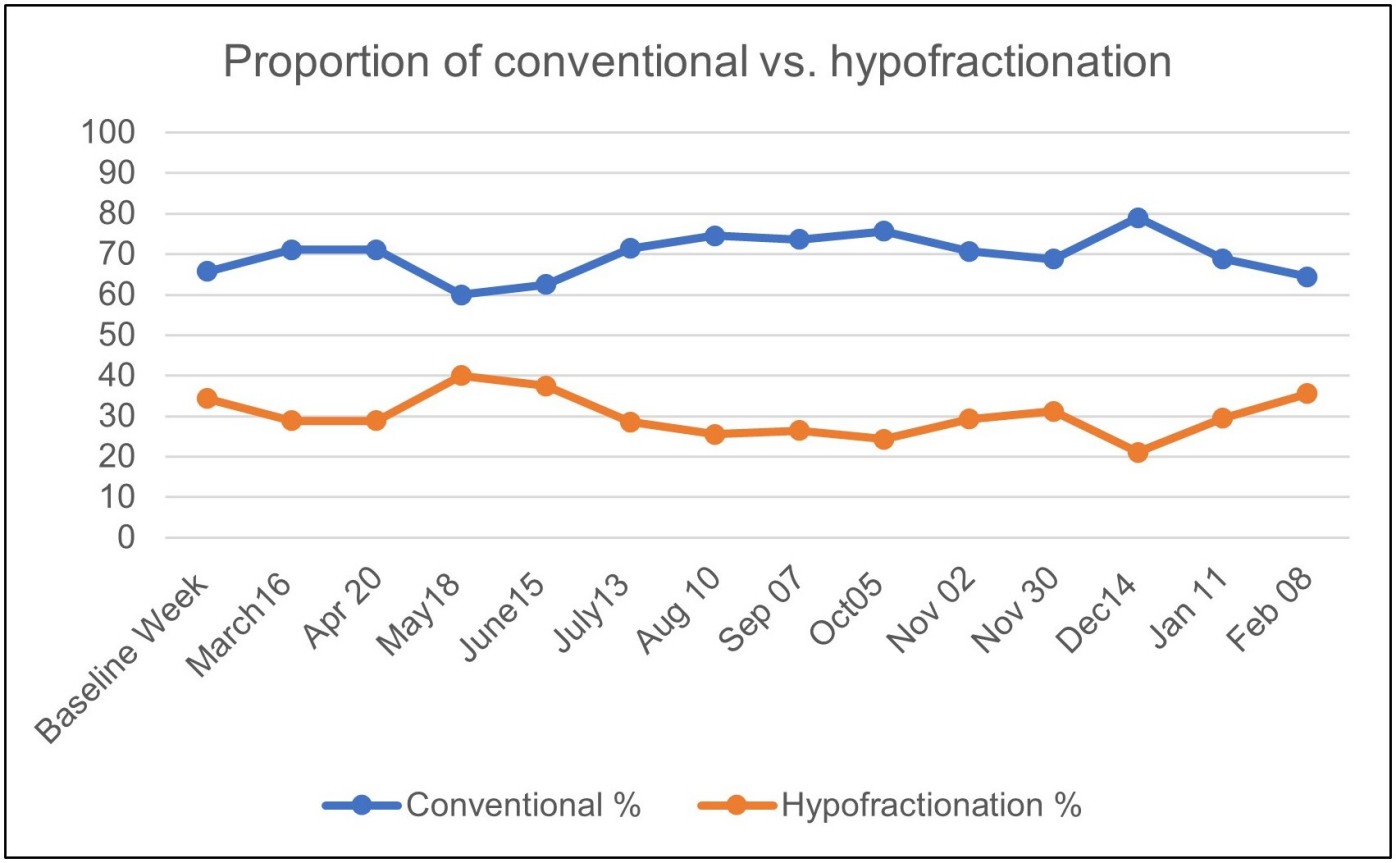
Changes in fractionation. Proportion of conventional fractionation versus hypofractionation (%) in the baseline week and throughout the pandemic.

After the COVID-19 outbreak, three institutions implemented hypofractionation in their program, and the proportion of moderate and ultrahypofractionation increased by 6% and 4%, respectively, in May and June 2020. In the subsequent months, the use of hypofractionation remained relatively stable and was applied in approximately 30% of the cases (Fig 5).

## Discussion

After the World Health Organization declared COVID-19 a pandemic on March 11, 2020, unprecedented challenges needed to be addressed globally. Measures included “stay at home” policies [16] and social distancing to “flatten the curve” [17], resulting in a decline in urological outpatient appointments by 81 to 100% according to a global survey by Teoh et al. [18]. The EAU recommended a delay of screening and diagnostic evaluation for PCa of up to six months and a biopsy without MRI if necessary [8]. As a surrogate for PCa screening, Ferrari et al. reported an order reduction of PSA test kits to 62% during the first Italian lockdown (March to May 2020) [19]. While a delayed PBx up to eight months after a suspicious MRI did not affect histopathological findings, Savin et al. underlined that patients with high-risk lesions and no previous biopsies should undergo earlier evaluation [20]. Our study also reports a decline in PBx during the first wave of the pandemic with a subsequent increase in the following months. However, the numbers never exceeded the baseline caseload as a potential indicator of an ongoing delay in screening and diagnosis. This phenomenon could also be observed in treatment numbers both in urology and in radiotherapy. Nevertheless, the cumulative patient number in 2020 surpassed 2019 even though the reported interventions per week did not reach baseline after the start of the pandemic, indicating a potential bias of the selected weeks as only five representative days per month were evaluated, actual total case numbers in the summer months might be higher. This finding is in line with a study from Sweden that showed a decrease in new PCa cases in 2020 but stable numbers of RPs and an increase in radiotherapy cases by 32% [21]. This rise in EBRT is a plausible consequence of the enhanced use of radiation therapy as an alternative to RP propagated by the EAU [8]. The avoidance of surgical therapy should decrease the number of hospitalized patients, freeing up anesthesia personnel and ventilator and intensive care capacities. However, deferred surgical cancer therapy results in substantial psychological distress for cancer patients [22]. The respondents of our German study could not confirm a treatment shift from RP to EBRT in either the first or second wave of the pandemic. Both therapeutic pathways were affected similarly with an initially decreased caseload, which was also reported by Domenig et al. [23].

Deferred treatment of low- and intermediate-risk PCa is safe for at least three months, while therapy of unfavorable pathological findings should be preferred [1, 7, 24]. The trend to delay low-risk patients was mainly reported by radiotherapists during the first wave. This group remained on a lower caseload level than intermediate- and high-risk cancer patients. Prioritization was less evident for RP, and surgical therapy was most common for intermediate-risk patients, followed by patients in the highest priority group.

The feasibility of surgical therapy in a pandemic setting has been demonstrated by an American study focusing on robotic RP [25]. Similarly, robotic centers in our cohort did not change their standard to an open approach despite several surgical societies initially recommending a restrained use of laparoscopic or robotic procedures due to a possibly increased risk of COVID-19 aerosol development [26].

Moderate hypofractionation is an internationally established concept in radiotherapy of PCa and shows equivalent outcomes compared to conventional EBRT with slightly higher gastrointestinal and genitourinary toxicity [3, 27, 28]. However, it is still not the standard of care in most participating institutions since it requires extensive and high-end image guidance, and the higher risk for toxicity must be discussed with the patient. To reduce virus exposure for patients and health care workers, a shift toward hypofractionation concepts was recommended by national and international societies for the duration of the COVID-19 outbreak, but hypofractionated schedules were newly introduced in only three additional participating radiotherapy departments. Nevertheless, most patients were treated with conventional EBRT, which contradicts findings from the UK with a higher percentage of moderate as well as ultrahypofractionation since the beginning of the pandemic [29]. Both private and public institutions were reluctant to implement shortened therapy schedules in the long term.

This study is not without limitations. Unfortunately, the response rate was below 40%, probably due to the number of time-consuming questions on exact patient numbers over 15 weeks. While two-week comparative data might not be long enough to accurately elucidate the exact trends, a longer preoperative period could have resulted in an even lower response rate. However, the questionnaires provided real-world evidence instead of estimations and delivered exclusive insight into treatment pathways for PCa from a German radiotherapy and urology point of view. The responses reflect the medical landscape in Germany, with 16 federal states and varying types of patient care (academic, nonacademic, private hospitals and practices).

Several lessons can be learned after more than one year of COVID-19: even though there was a decline in the diagnosis and therapy of PCa, the overall numbers in 2020 increased compared to the preceding year. Thus, the fear of a wave of missed prostate cancer patients may be eased in Germany. At the same time, these data confirm that there is plenty of hospital capacity in Germany that did not have to be devoted in great part to COVID-19 care. Second, an even more modest decline in surgical patient care during the second wave of COVID-19 was observed. RP is a highly standardized procedure with a low complication rate and a short length of hospital stay [30]. While surgical capacities were reduced more significantly during spring 2020, prioritization seemed to focus on oncological procedures with a minor risk of ICU treatment during the second wave. Third, even though it is a well-established concept, the widespread use of hypofractionation is still in its infancy, and extensive use may further reduce the number of radiation office visits without an increased risk for oncological outcomes [27, 28].

The COVID-19 pandemic will probably continue to play a major role in the foreseeable future with upcoming new virus variants, changing efficacy of vaccines and persisting numbers of unvaccinated patients. It is a soothing thought that the full potential of the recommended measures was not reached.

## Conclusion

Diagnosis and therapy of PCa was affected in the first wave of the pandemic, with a subsequent recovery in the following months leading to a higher total number of treated patients in 2020 compared to the preceding year. We found a heterogeneous implementation of the recommendations regarding patient prioritization in local cancer treatment and fractionation regimes in radiotherapy. This highlights unemployed resources for the next peaks of the pandemic.

## Supporting information

S1 Dataset (PDF)
