# Supplementary material for: Lessons learned after one year of COVID-19 from a urologist and radiotherapist view: A German survey on prostate cancer diagnosis and treatment
Source: PLoS One. 2022 Jun 14;17(6):e0269827. doi: 10.1371/journal.pone.0269827 (PMC9197019; doi:10.1371/journal.pone.0269827)
Supplement: S1 Dataset — (PDF) [file pone.0269827.s001.pdf]

Nummer	Gesamtzahl der Prostatakarzinon	Gesamtzahl der Pro	Fach
1	40	63	0
2	40	58	0
3	196	229	0
4	86	78	0
5	152	121	0
6	29	46	0
7	98	87	0
8	87	61	0
9	36	57	0
10	113	112	0
11	11	10	0
12	86	82	0
13	175	188	0
14	68	74	0
15	67	79	0
16	67	65	0
17	51	75	0
18	61	57	0
19	142	148	0
20	165	168	0
21	119	121	1
22	1164	1430	1
23	438	495	1
24	250	253	1
25	240	250	1
26	580	551	1
27	80	75	1
28	193	274	1
29	154	196	1
30	400	480	1
31	123	130	1
32	70	65	1
33	167	143	1
34	186	197	1
35	86	54	1
36	165	162	1
37	62	71	1
38	48	51	1
39	42	50	1
40	290	302	1
41	46	39	1
42	43	55	1
43	66	96	1
44	159	128	1
45	141	125	1
46	258	252	1
47	220	182	1

Baseline 1 a) Anzahl Low I	Baseline 1 b) Anzahl Intern	Baseline 1 c) Anzahl High	Baseline 1 d) Anzahl lokal
0	5	0	0
2	4	2	0
2	6	2	0
0	2	1	0
2	3	3	0
0	0	1	0
0	2	1	0
0	0	1	0
0	0	2	0
0	1	2	0
0	0	0	0
1	2	1	0
2	1	0	0
0	0	4	0
0	1	0	0
0	2	0	0
0	0	2	0
2	3	2	0
0	1	3	0
0	2	2	0
0	1	1	0
5	16	12	0
0	12	3	0
1	4	2	0
1	4	3	0
4	8	6	0
1	1	1	0
0	4	0	0
0	3	1	0
0	6	6	0
1	2	2	0
0	2	2	0
1	4	1	0
1	1	0	0
0	0	2	0
1	0	3	0
0	2	1	0
0	0	0	0
0	0	0	0
1	4	0	0
0	0	1	0
0	1	1	0
1	1	1	0
3	2	1	0
0	1	1	0
2	5	1	0
1	2	3	0

Baseline 1 Anzahl der beh	Baseline 1 Wenden Sie Hy	Baseline 1 Anzahl der Pati	Baseline 1 Anzahl der Pati
3	2	0	2
4	1	4	0
8	1	2	0
0	3	1	2
2	1	6	0
1	0	0	0
3	0	0	0
0	1	1	0
0	1	2	0
2	1	1	0
0	0	0	0
3	1	1	0
3	0	0	0
4	0	0	0
1	0	0	0
2	0	0	0
2	0	0	0
7	0	0	0
4	0	0	0
4	0	0	0

Baseline 2 a) Anzahl Low I	Baseline 2 b) Anzahl Intern	Baseline 2 c) Anzahl High	Baseline 2 d) Anzahl lokal
0	3	2	0
1	3	2	0
1	7	4	0
0	1	0	0
3	1	2	0
0	0	2	0
0	2	1	0
1	1	0	0
0	1	1	0
0	3	0	0
0	0	1	0
0	0	4	0
0	0	0	0
0	1	0	0
1	0	1	0
0	1	0	0
0	0	1	0
0	5	0	0
0	2	0	0
0	0	3	0
0	1	1	0
6	12	11	0
0	7	4	0
0	5	2	0
0	5	2	0
2	10	1	0
1	1	1	0
2	5	1	0
0	3	2	0
1	5	9	0
1	1	2	0
0	1	1	0
1	1	3	0
1	2	2	0
1	1	0	0
1	2	1	0
0	1	2	0
1	0	0	0
0	1	0	0
2	3	4	0
1	1	0	0
1	1	0	0
0	2	1	0
0	2	2	0
2	0	1	0
0	3	5	0
1	2	2	0

Baseline 2 Anzahl der beh	Baseline 2 Wenden Sie Hy	Baseline 2 Anzahl der Pati	Baseline 2 Anzahl der Pati
3	2	0	2
3	1	3	0
5	1	7	0
0	1	1	0
3	1	3	0
2	0	0	0
3	0	0	0
0	1	2	0
0	1	2	0
1	1	2	0
0	1	1	0
2	1	2	0
0	0	0	0
1	0	0	0
2	0	0	0
1	0	0	0
1	0	0	0
5	0	0	0
2	0	0	0
3	0	0	0

March16 a) Anzahl Low Ri	March16 b) Anzahl Interme	March16 c) Anzahl High Ri	March16 d) Anzahl lokal fo
0	3	0	0
0	3	1	0
1	3	1	0
0	0	1	0
0	0	1	0
0	0	1	0
0	2	1	0
0	1	0	0
0	0	0	0
0	1	0	0
0	0	0	0
0	1	1	0
0	2	1	0
1	3	1	0
1	0	1	0
0	1	0	0
0	0	0	0
1	3	2	0
0	2	0	0
0	1	3	0
0	1	1	0
4	19	6	0
2	1	4	0
0	5	2	0
1	3	3	0
1	6	2	0
0	1	1	0
0	4	4	0
1	4	2	0
0	2	12	0
0	1	2	0
0	0	0	0
0	2	1	0
1	3	1	0
0	0	0	0
2	2	0	0
0	0	2	0
0	0	0	0
0	0	0	0
4	2	2	0
0	1	0	0
0	1	1	0
0	2	0	0
1	1	1	0
1	5	1	0
1	6	2	0
0	3	4	0

March16 Anzahl der behar	March16 Wenden Sie Hyp	March16 Anzahl der Patier	March16 Anzahl der Patier
1	1	2	0
2	1	2	0
3	1	2	0
0	1	1	0
0	1	1	0
1	0	0	0
2	1	1	0
0	1	1	0
0	0	0	0
0	1	1	0
0	0	0	0
1	1	1	0
2	1	1	0
5	0	0	0
2	0	0	0
1	0	0	0
0	0	0	0
6	0	0	0
2	0	0	0
4	0	0	0

April20 a) Anzahl Low Risk	April20 b) Anzahl Intermed	April20 c) Anzahl High Risk	April20 d) Anzahl lokal fort
0	2	0	0
1	4	3	0
0	3	0	0
0	0	2	0
0	2	1	0
0	0	0	0
0	0	0	0
0	0	1	0
0	1	0	0
1	0	1	0
0	0	0	0
0	1	3	0
1	1	4	0
0	2	0	0
1	1	3	0
0	0	0	0
0	0	2	0
1	2	0	0
0	0	0	0
0	0	1	0
2	1	1	0
4	17	10	0
0	7	5	0
2	2	1	0
0	5	2	0
0	8	4	0
0	1	1	0
0	0	2	0
0	3	3	0
1	5	7	0
1	1	2	0
0	3	0	0
2	2	1	0
2	1	3	0
0	0	2	0
1	3	1	0
0	0	0	0
0	0	0	0
0	0	0	0
0	5	1	0
0	0	0	0
0	3	2	0
0	1	2	0
0	0	1	0
1	2	2	0
1	2	1	0
0	3	2	0

April20 Anzahl der behand	April20 Wenden Sie Hypof	April20 Anzahl der Patiente	April20 Anzahl der Patiente
2	0	0	0
4	1	4	0
2	1	1	0
0	3	1	1
1	1	2	0
0	0	0	0
0	0	0	0
0	1	1	0
0	1	1	0
2	0	0	0
0	0	0	0
2	1	2	0
6	0	0	0
2	0	0	0
5	0	0	0
0	0	0	0
2	0	0	0
3	0	0	0
0	0	0	0
1	0	0	0

May18 a) Anzahl Low Risk	May18 b) Anzahl Intermed	May18 c) Anzahl High Risk	May18 d) Anzahl lokal fortg
0	2	2	0
1	4	2	0
0	4	1	0
0	0	0	0
1	1	2	0
0	1	0	0
0	1	0	0
0	0	1	0
0	0	0	0
0	0	1	0
0	0	0	0
1	0	1	0
0	0	0	0
0	2	0	0
1	0	0	0
0	0	1	0
0	0	0	0
1	2	2	0
0	0	0	0
0	0	0	0
0	1	1	0
3	19	5	0
0	4	3	0
0	3	0	0
0	2	1	0
0	6	2	0
1	1	0	0
0	0	2	0
1	1	2	0
2	8	8	0
0	1	0	0
0	0	0	0
0	1	2	0
0	0	0	0
0	0	0	0
0	2	0	0
0	0	0	0
1	1	0	0
0	0	0	0
2	1	4	0
0	0	1	0
0	0	0	0
0	0	0	0
0	1	0	0
0	1	2	0
0	6	0	0
0	1	1	0

May18 Anzahl der behand	May18 Wenden Sie Hypofi	May18 Anzahl der Patiente	May18 Anzahl der Patiente
3	2	0	1
4	1	3	0
3	1	2	0
0	0	0	0
1	1	3	0
1	0	0	0
0	1	1	0
0	1	1	0
0	0	0	0
0	1	1	0
0	0	0	0
0	1	2	0
0	0	0	0
2	0	0	0
1	0	0	0
1	0	0	0
0	0	0	0
5	0	0	0
0	0	0	0
0	0	0	0

June15 a) Anzahl Low Risl	June15 b) Anzahl Intermed	June15 c) Anzahl High Ris	June15 d) Anzahl lokal fort
0	1	1	0
1	5	2	0
1	3	2	0
0	2	0	0
0	0	0	0
0	1	2	0
0	1	1	0
0	1	1	0
0	4	1	0
2	2	0	0
0	0	0	0
0	2	1	0
0	1	1	0
0	1	1	0
0	0	2	0
0	0	0	0
0	0	2	0
0	2	1	0
0	0	0	0
1	2	5	0
0	1	1	0
6	17	11	0
0	7	4	0
0	3	3	0
0	0	0	0
1	9	3	0
0	1	1	0
0	3	4	0
1	1	2	0
0	6	6	0
1	2	1	0
0	0	0	0
0	2	0	0
1	2	0	0
0	1	0	0
0	0	1	0
0	1	0	0
1	0	0	0
0	2	0	0
3	1	1	0
0	0	0	0
0	3	0	0
0	2	0	0
1	1	1	0
0	0	3	0
0	7	1	0
1	2	2	0

June15 Anzahl der behand	June15 Wenden Sie Hypot	June15 Anzahl der Patient	June15 Anzahl der Patient
2	0	0	0
4	1	4	0
2	1	4	0
0	2	0	2
0	0	0	0
2	1	1	0
1	1	1	0
0	1	2	0
0	1	5	0
2	1	2	0
0	0	0	0
3	0	0	0
2	0	0	0
2	0	0	0
2	0	0	0
0	0	0	0
2	0	0	0
3	0	0	0
0	0	0	0
8	0	0	0

July13 a) Anzahl Low Risk	July13 b) Anzahl Intermedi	July13 c) Anzahl High Risk	July13 d) Anzahl lokal fortg
0	4	0	0
0	4	2	0
0	2	0	0
0	1	0	0
0	1	2	0
0	0	1	0
0	0	1	0
0	0	0	0
0	0	2	0
1	0	1	0
0	0	1	0
0	3	0	0
1	0	0	0
0	0	2	0
0	0	0	0
0	0	0	0
0	2	0	0
1	0	2	0
0	2	0	0
2	3	8	0
0	2	1	0
2	15	12	0
1	3	4	0
0	0	0	0
1	3	3	0
4	5	4	0
1	0	1	0
3	2	1	0
2	4	0	0
1	5	5	0
0	1	1	0
0	1	1	0
0	1	1	0
2	1	1	0
0	0	0	0
0	0	0	0
0	2	0	0
0	0	0	0
0	2	0	0
0	6	1	0
0	0	0	0
0	2	0	0
0	1	1	0
1	1	0	0
0	1	1	0
0	4	2	0
0	1	1	0

July13 Anzahl der behande	July13 Wenden Sie Hypofr	July13 Anzahl der Patiente	July13 Anzahl der Patiente
3	1	1	0
3	1	3	0
1	1	1	0
0	1	1	0
0	1	3	0
0	1	1	0
1	0	0	0
0	0	0	0
0	1	2	0
1	1	1	0
0	1	1	0
3	0	0	0
1	0	0	0
2	0	0	0
0	0	0	0
0	0	0	0
2	0	0	0
3	0	0	0
2	0	0	0
13	0	0	0

Aug10 a) Anzahl Low Risk	Aug10 b) Anzahl Intermedi	Aug10 c) Anzahl High Risk	Aug10 d) Anzahl lokal fortg
0	3	0	0
1	3	1	0
1	1	2	0
0	1	0	0
0	1	2	0
1	3	0	0
0	0	1	0
0	0	0	0
0	2	0	0
0	2	1	0
0	0	0	0
0	1	1	0
0	0	3	0
0	0	0	0
0	1	1	0
0	1	0	0
0	0	0	0
0	0	0	0
0	2	2	0
2	3	8	0
0	2	1	0
6	24	4	0
0	5	4	0
1	4	0	0
0	5	3	0
2	3	8	0
0	1	1	0
1	6	1	0
0	1	1	0
0	7	4	0
0	0	3	0
0	2	1	0
0	3	0	0
1	1	1	0
0	0	0	0
0	1	1	0
0	1	1	0
2	0	0	0
0	1	0	0
0	3	2	0
1	0	0	0
0	0	0	0
0	0	2	0
2	0	0	0
1	1	1	0
2	2	3	0
0	2	3	0

Aug10 Anzahl der behande	Aug10 Wenden Sie Hypofr	Aug10 Anzahl der Patiente	Aug10 Anzahl der Patiente
2	2	0	1
3	1	2	0
2	1	2	0
0	1	1	0
0	1	3	0
4	0	0	0
1	0	0	0
0	0	0	0
0	1	2	0
1	1	2	0
0	0	0	0
2	0	0	0
3	0	0	0
0	0	0	0
2	0	0	0
1	0	0	0
0	0	0	0
0	0	0	0
4	0	0	0
13	0	0	0

Sept07 a) Anzahl Low Risk	Sept07 b) Anzahl Intermed	Sept07 c) Anzahl High Risk	Sept07 d) Anzahl lokal fort
0	3	0	0
1	6	3	0
0	2	0	0
0	0	0	0
0	0	0	0
0	1	0	0
0	0	2	0
0	0	0	0
0	0	1	0
0	1	3	0
1	0	0	0
1	2	2	0
2	1	4	0
0	0	0	0
0	1	0	0
0	3	0	0
0	1	0	0
1	2	0	0
0	1	2	0
0	1	5	0
0	2	1	0
5	17	13	0
0	4	1	0
0	3	1	0
0	0	0	0
2	5	3	0
1	0	1	0
0	4	4	0
0	1	3	0
0	5	10	0
0	0	1	0
0	0	0	0
0	0	1	0
1	0	0	0
0	1	0	0
0	1	2	0
0	0	1	0
0	0	0	0
0	1	1	0
1	3	2	0
1	0	0	0
0	0	0	0
0	1	1	0
1	1	1	0
1	1	1	0
2	0	0	0
1	2	2	0

Sept07 Anzahl der behand	Sept07 Wenden Sie Hypof	Sept07 Anzahl der Patiente	Sept07 Anzahl der Patiente
1	2	0	2
5	3	4	1
1	1	1	0
0	0	0	0
0	0	0	0
0	1	1	0
1	1	1	0
0	0	0	0
0	1	1	0
2	1	2	0
0	1	1	0
5	0	0	0
7	0	0	0
0	0	0	0
1	0	0	0
3	0	0	0
1	0	0	0
3	0	0	0
3	0	0	0
6	0	0	0

Oct05 a) Anzahl Low Risk	Oct05 b) Anzahl Intermedi	Oct05 c) Anzahl High Risk	Oct05 d) Anzahl lokal fortg
0	4	0	0
0	2	2	0
1	3	1	0
0	1	0	0
0	0	0	0
0	0	0	0
0	0	2	0
0	0	0	0
0	0	0	0
0	1	1	0
0	0	0	0
0	0	4	0
0	1	2	0
0	1	0	0
0	0	0	0
0	0	0	0
0	1	0	0
0	1	2	0
0	2	1	0
0	1	3	0
0	4	1	0
10	14	10	0
0	4	6	0
1	4	2	0
1	3	1	0
2	3	7	0
0	1	1	0
0	4	2	0
1	2	1	0
2	5	5	0
0	1	0	0
0	1	0	0
0	0	1	0
1	2	1	0
0	1	0	0
1	0	1	0
0	1	1	0
0	0	0	0
0	0	0	0
0	1	2	0
0	1	0	0
0	1	0	0
1	1	0	0
2	1	2	0
0	2	2	0
1	2	2	0
0	2	2	0

Oct05 Anzahl der behande	Oct05 Wenden Sie Hypofr	Oct05 Anzahl der Patienten	Oct05 Anzahl der Patienten
3	2	0	1
2	1	2	0
2	1	3	0
0	1	1	0
0	0	0	0
0	0	0	0
2	0	0	0
0	0	0	0
0	0	0	0
1	1	1	0
0	0	0	0
4	0	0	0
2	1	1	0
1	0	0	0
0	0	0	0
0	0	0	0
1	0	0	0
3	0	0	0
3	0	0	0
4	0	0	0

Nov02 a) Anzahl Low Risk	Nov02 b) Anzahl Intermedi	Nov02 c) Anzahl High Risk	Nov02 d) Anzahl lokal fortg
0	1	0	0
0	2	2	0
1	1	1	0
0	2	0	0
0	0	2	0
0	1	0	0
0	1	1	0
1	3	0	0
0	0	0	0
0	1	0	0
0	0	0	0
1	1	1	0
0	1	0	0
0	1	0	0
0	1	0	0
0	0	0	0
0	0	0	0
2	2	1	0
1	0	2	0
1	3	3	0
0	2	1	0
11	21	4	0
1	6	0	0
1	1	1	0
0	2	2	0
2	7	2	0
1	1	1	0
2	3	2	0
0	4	2	0
0	8	7	0
0	0	0	0
0	0	2	0
2	3	0	0
1	1	0	0
0	1	0	0
2	2	0	0
0	2	0	0
1	0	0	0
0	2	0	0
2	2	0	0
0	0	0	0
0	1	0	0
0	2	0	0
1	1	2	0
0	1	0	0
1	4	0	0
0	2	1	0

Nov02 Anzahl der behande	Nov02 Wenden Sie Hypofr	Nov02 Anzahl der Patiente	Nov02 Anzahl der Patiente
1	0	0	0
2	1	2	0
1	1	2	0
0	1	2	0
1	1	1	0
1	0	0	0
2	0	0	0
0	1	4	0
0	0	0	0
1	0	0	0
0	0	0	0
3	0	0	0
0	1	1	0
1	0	0	0
1	0	0	0
0	0	0	0
0	0	0	0
5	0	0	0
3	0	0	0
7	0	0	0

Nov30 a) Anzahl Low Risk	Nov30 b) Anzahl Intermedi	Nov30 c) Anzahl High Risk	Nov30 d) Anzahl lokal fortg
0	3	0	0
0	4	2	0
1	0	1	0
1	1	0	0
0	0	1	0
0	0	0	0
0	1	2	0
0	1	0	0
0	0	0	0
0	0	2	0
0	0	0	0
0	3	1	0
1	1	0	0
0	0	0	0
0	1	0	0
0	1	1	0
0	0	0	0
0	1	1	0
0	0	0	0
0	0	1	0
0	2	0	0
5	18	7	0
0	4	4	0
0	3	1	0
1	4	1	0
1	7	5	0
1	1	1	0
1	1	2	0
0	5	1	0
0	7	7	0
0	0	2	0
0	2	0	0
0	1	1	0
1	3	1	0
1	0	0	0
0	1	1	0
0	0	0	0
0	1	0	0
0	1	1	0
2	3	1	0
0	0	0	0
0	0	0	0
0	1	0	0
1	2	0	0
1	1	1	0
1	4	0	0
0	0	3	0

Nov30 Anzahl der behande	Nov30 Wenden Sie Hypofr	Nov30 Anzahl der Patiente	Nov30 Anzahl der Patiente
3	0	0	0
3	1	3	0
1	1	1	0
0	3	1	1
0	1	1	0
0	0	0	0
3	0	0	0
0	1	1	0
0	0	0	0
1	1	1	0
0	0	0	0
3	1	1	0
2	0	0	0
0	0	0	0
1	0	0	0
2	0	0	0
0	0	0	0
2	0	0	0
0	0	0	0
1	0	0	0

Dec14 a) Anzahl Low Risk	Dec14 b) Anzahl Intermedi	Dec14 c) Anzahl High Risk	Dec14 d) Anzahl lokal fortg
0	1	0	0
1	3	2	0
0	1	0	0
0	0	0	0
0	0	0	0
0	1	1	0
0	0	0	0
0	0	0	0
0	0	0	0
0	0	1	0
0	0	0	0
0	0	1	0
0	0	0	0
0	0	0	0
1	2	0	0
0	0	0	0
0	1	1	0
0	0	2	0
0	0	0	0
0	0	0	0
0	0	0	0
5	16	7	0
0	5	4	0
0	2	1	0
1	2	1	0
1	3	4	0
2	0	0	0
1	1	4	0
0	1	2	0
0	10	5	0
3	1	0	0
0	0	1	0
1	1	1	0
1	1	0	0
0	1	0	0
3	0	0	0
0	0	0	0
2	1	0	0
0	0	0	0
2	3	1	0
0	0	2	0
0	2	0	0
0	2	0	0
0	0	1	0
0	0	0	0
0	4	1	0
0	2	2	0

Dec14 Anzahl der behande	Dec14 Wenden Sie Hypofr	Dec14 Anzahl der Patiente	Dec14 Anzahl der Patiente
1	0	0	0
3	3	2	1
0	1	1	0
0	0	0	0
0	0	0	0
2	0	0	0
0	0	0	0
0	0	0	0
0	0	0	0
1	0	0	0
0	0	0	0
1	0	0	0
0	0	0	0
0	0	0	0
3	0	0	0
0	0	0	0
2	0	0	0
2	0	0	0
0	0	0	0
0	0	0	0

Jan11 a) Anzahl Low Risk	Jan11 b) Anzahl Intermedi	Jan11 c) Anzahl High Risk	Jan11 d) Anzahl lokal fortg
0	2	0	0
1	2	1	0
2	4	2	0
0	2	1	0
0	0	5	0
1	1	0	0
0	1	2	0
0	0	0	0
0	0	0	0
0	0	2	0
0	0	1	0
0	2	1	0
1	0	3	0
1	1	0	0
1	0	1	0
0	0	2	0
0	0	0	0
2	1	0	0
0	3	3	0
1	3	5	0
1	2	1	0
2	21	12	0
0	7	4	0
0	6	2	0
0	2	1	0
6	11	4	0
0	1	1	0
1	4	1	0
0	2	1	0
0	9	4	0
1	0	2	0
0	0	1	0
1	4	1	0
1	0	0	0
0	1	0	0
0	2	2	0
0	0	1	0
2	0	1	0
0	0	0	0
1	6	1	0
0	0	0	0
0	2	0	0
0	2	0	0
0	4	1	0
1	1	2	0
1	5	2	0
1	1	2	0

Jan11 Anzahl der behande	Jan11 Wenden Sie Hypofr	Jan11 Anzahl der Patiente	Jan11 Anzahl der Patiente
1	2	0	1
2	1	2	0
4	1	4	0
0	1	3	0
1	1	4	0
1	1	1	0
2	1	1	0
0	0	0	0
0	0	0	0
1	1	1	0
0	1	1	0
3	0	0	0
3	0	0	0
2	0	0	0
2	0	0	0
2	0	0	0
0	0	0	0
3	0	0	0
6	0	0	0
9	0	0	0

Feb08 a) Anzahl Low Risk	Feb08 b) Anzahl Intermedi	Feb08 c) Anzahl High Risk	Feb08 d) Anzahl lokal fo
0	3	1	0
0	3	1	0
1	1	0	0
0	2	3	0
0	0	1	0
0	0	1	0
0	3	2	0
0	1	0	0
0	0	1	0
0	0	1	0
0	0	0	0
0	1	2	0
0	0	0	0
0	0	0	0
0	0	1	0
0	1	1	0
0	2	0	0
2	2	0	0
0	1	3	0
0	3	1	0
0	0	4	0
5	20	9	0
0	8	2	0
0	4	2	0
0	4	1	0
2	6	1	0
0	2	0	0
1	4	1	0
1	3	1	0
0	4	11	0
2	1	0	0
0	1	0	0
0	2	1	0
0	2	2	0
1	0	0	0
0	1	1	0
0	0	0	0
1	0	0	0
0	0	0	0
1	1	2	0
0	1	0	0
0	2	1	0
0	1	1	0
0	3	0	0
1	2	0	0
0	2	3	0
0	1	2	0

Feb08 Anzahl der behande	Feb08 Wenden Sie Hypofr	Feb08 Anzahl der Patiente	Feb08 Anzahl der Patiente
2	2	0	2
2	1	2	0
1	1	1	0
0	1	5	0
0	1	1	0
0	1	1	0
3	1	2	0
0	1	1	0
0	1	1	0
1	0	0	0
0	0	0	0
3	0	0	0
0	0	0	0
0	0	0	0
1	0	0	0
2	0	0	0
2	0	0	0
4	0	0	0
4	0	0	0
4	0	0	0

BL_1 Wie viele Prostatasta	BL_1 a) Anzahl Low Risk P	BL_1 b) Anzahl Intermedia	BL_1 c) Anzahl High Risk P
9	0	1	1
7	5	16	12
10	0	12	3
9	1	4	2
6	1	4	3
15	4	8	6
5	1	1	1
9	0	4	0
9	0	3	1
15	0	6	6
4	1	2	2
4	0	2	2
4	1	4	1
7	1	1	0
4	0	0	2
5	1	0	3
2	0	2	1
5	0	0	0
3	0	0	0
6	1	4	0
4	0	0	1
2	0	1	1
7	1	1	1
7	3	2	1
5	0	1	1
12	2	5	1
9	1	2	3

BL_1 d) Anzahl lokal fortge	BL_1 Wie viele offene radi	BL_1 Wie viele roboterass	BL_1 Wie viele laparoskop
0	0	2	0
0	0	33	0
0	13	2	0
0	0	7	0
0	0	8	0
0	5	13	0
0	0	3	0
0	1	3	0
0	0	4	0
0	0	12	0
0	0	5	0
0	0	4	0
0	0	6	0
0	0	2	0
0	0	2	0
0	0	4	0
0	1	0	2
0	0	0	0
0	0	0	0
0	0	5	0
0	0	0	1
0	0	2	0
0	1	2	0
0	0	6	0
0	0	2	0
0	3	5	0
0	0	6	0

BL_1 Wie viele fokale The	BL_1 Ich möchte den Frag	BL_2 Wie viele Prostatasta	BL_2 a) Anzahl Low Risk F
0		7	0
0		5	6
0	Ja, ich möchte das Formul	9	0
1		8	0
0		5	0
0		14	2
0		5	1
0	Nein, ich möchte die nächs	6	2
2		3	0
0		18	1
0		3	1
0		3	0
0		10	1
0		6	1
0		6	1
0		4	1
0		4	0
0		1	1
0	Nein, ich möchte die nächs	4	0
0	1	6	2
11		5	1
0		2	1
0		4	0
0		4	0
0		1	2
0		5	0
0		10	1

BL_2 b) Anzahl Intermedia	BL_2 c) Anzahl High Risk	BL_2 d) Anzahl lokal fortge	BL_2 Wie viele offene radi
1	1	0	0
12	11	0	0
7	4	0	9
5	2	0	0
5	2	0	0
10	1	0	2
1	1	0	0
5	1	0	2
3	2	0	0
5	9	0	0
1	2	0	0
1	1	0	1
1	3	0	0
2	2	0	0
1	0	0	1
2	1	0	0
1	2	0	1
0	0	0	0
1	0	0	0
3	4	0	0
1	0	0	0
1	0	0	0
2	1	0	0
2	2	0	0
0	1	0	0
3	5	0	5
2	2	0	0

BL_2 Wie viele roboterass	BL_2 Wie viele laparoskop	BL_2 Wie viele fokale The	BL_2 Ich möchte den Frag
2	0	0	
29	0	0	
2	0	0	Nein, ich möchte die nächs
7	0	0	
7	0	0	
11	0	0	
3	0	0	
6	0	0	Nein, ich möchte die nächs
5	0	0	
15	0	0	
4	0	0	
1	0	0	
5	0	0	
5	0	0	
1	0	0	
4	0	0	
0	2	0	
1	0	0	
1	0	0	Nein, ich möchte die nächs
9	0	0	1
0	2	1	
2	0	0	
3	0	0	
4	0	0	
3	0	0	
3	0	0	
5	0	0	

March16 Wie viele Prostat	March16 a) Anzahl Low Ri	March16 b) Anzahl Interme	March16 c) Anzahl High R
5	0	1	1
12	4	19	6
7	2	1	4
8	0	5	2
4	1	3	3
8	1	6	2
2	0	1	1
6	0	4	4
11	1	4	2
5	0	2	12
2	0	1	2
0	0	0	0
4	0	2	1
1	1	3	1
1	0	0	0
2	2	2	0
1	0	0	2
1	0	0	0
0	0	0	0
6	4	2	2
4	0	1	0
0	0	1	1
5	0	2	0
4	1	1	1
3	1	5	1
12	1	6	2
9	0	3	4

March16 d) Anzahl lokal fo	March16 Wie viele offene	March16 Wie viele roboter	March16 Wie viele laparos
0	0	2	0
0	0	29	0
0	7	0	0
0	0	7	0
0	0	7	0
0	0	9	0
0	0	2	0
0	0	8	0
0	1	6	0
0	0	14	0
0	0	3	0
0	0	0	0
0	0	3	0
0	0	5	0
0	0	0	0
0	0	4	0
0	1	0	1
0	0	0	0
0	0	0	0
0	0	8	0
0	0	0	1
0	0	2	0
0	0	2	0
0	0	3	0
0	0	7	0
0	6	3	0
0	0	7	0

March16 Wie viele fokale T	March16 Ich möchte den F	April20 Wie viele Prostatas	April20 a) Anzahl Low Risk
0		6	2
0		3	4
0	Nein, ich möchte die nächs	6	0
0		7	2
0		4	0
0		16	0
0		3	0
0	Nein, ich möchte die nächs	7	0
0		4	0
		12	1
0		1	1
0		0	0
0		4	2
0		1	2
0		0	0
0		0	1
0		3	0
0		3	0
0	Nein, ich möchte die nächs	4	0
0	1	7	0
2		6	0
0		1	0
0		5	0
0		0	0
0		1	1
0		7	1
0		8	0

April20 b) Anzahl Intermed	April20 c) Anzahl High Risl	April20 d) Anzahl lokal fort	April20 Wie viele offene ra
1	1	0	0
17	10	0	0
7	5	0	12
2	1	0	0
5	2	0	0
8	4	0	0
1	1	0	0
0	2	0	2
3	3	0	0
5	7	0	0
1	2	0	0
3	0	0	0
2	1	0	0
1	3	0	0
0	2	0	0
3	1	0	0
0	0	0	0
0	0	0	0
0	0	0	0
5	1	0	0
0	0	0	0
3	2	0	0
1	2	0	1
0	1	0	0
2	2	0	0
2	1	0	2
3	2	0	0

April20 Wie viele roboteras	April20 Wie viele laparosko	April20 Wie viele fokale Th	April20 Ich möchte den Fra
4	0	0	
31	0	0	
0	0	0	Nein, ich möchte die nächs
5	0	0	
7	0	0	
12	0	0	
2	0	0	
0	0	0	Nein, ich möchte die nächs
6	0	0	
13	0	0	
4	0	0	
3	0	0	
5	0	0	
6	0	0	
2	0	0	
5	0	0	
0	0	0	
0	0	0	
0	0	0	Nein, ich möchte die nächs
6	0	2	1
0	0	5	
5	0	0	
2	0	0	
1	0	0	
5	0	0	
2	0	0	
5	0	0	

May18 Wie viele Prostatas	May18 a) Anzahl Low Risk	May18 b) Anzahl Intermed	May18 c) Anzahl High Risk
1	0	1	1
6	3	19	5
7	0	4	3
6	0	3	0
5	0	2	1
14	0	6	2
2	1	1	0
1	0	0	2
8	1	1	2
25	2	8	8
1	0	1	0
0	0	0	0
4	0	1	2
3	0	0	0
1	0	0	0
3	0	2	0
6	0	0	0
2	1	1	0
3	0	0	0
6	2	1	4
4	0	0	1
0	0	0	0
1	0	0	0
2	0	1	0
3	0	1	2
5	0	6	0
5	0	1	1

May18 d) Anzahl lokal fortg	May18 Wie viele offene rad	May18 Wie viele roboteras	May18 Wie viele laparosko
0	0	2	0
0	0	27	0
0	5	2	0
0	0	3	0
0	0	3	0
0	0	8	0
0	0	2	0
0	2	0	0
0	0	4	0
0	0	18	0
0	0	1	0
0	0	0	0
0	0	3	0
0	0	0	0
0	0	0	0
0	0	2	0
0	0	0	0
0	0	2	0
0	0	0	0
0	0	7	0
0	0	0	1
0	0	0	0
0	0	0	0
0	0	1	0
0	0	3	0
0	2	4	0
0	0	2	0

May18 Wie viele fokale Th	May18 Ich möchte den Fra	June15 Wie viele Prostata	June15 a) Anzahl Low Risl
0		6	0
0		11	6
0	Nein, ich möchte die nächs	3	0
0		7	0
0		6	0
0		13	1
0		2	0
0	Nein, ich möchte die nächs	7	0
0		2	1
0		18	0
0		2	1
0		0	0
0		3	0
0		8	1
0		2	0
0		4	0
0		4	0
0		2	1
0	Nein, ich möchte die nächs	5	0
0	1	3	3
12		1	0
0		0	0
0		3	0
0		3	1
0		1	0
0		8	0
0		5	1

June15 b) Anzahl Intermed	June15 c) Anzahl High Ris	June15 d) Anzahl lokal fort	June15 Wie viele offene ra
1	1	0	0
17	11	0	0
7	4	0	10
3	3	0	0
0	0	0	0
9	3	0	0
1	1	0	0
3	4	0	2
1	2	0	0
6	6	0	0
2	1	0	0
0	0	0	0
2	0	0	0
2	0	0	0
1	0	0	1
0	1	0	0
1	0	0	0
0	0	0	0
2	0	0	1
1	1	0	0
0	0	0	0
3	0	0	0
2	0	0	0
1	1	0	0
0	3	0	0
7	1	0	4
2	2	0	0

June15 Wie viele roboteras	June15 Wie viele laparosk	June15 Wie viele fokale Th	June15 Ich möchte den Fra
2	0	0	
34	0	0	
1	0	0	Nein, ich möchte die nächs
6	0	0	
0	0	0	
13	0	0	
2	0	0	
5	0	0	Nein, ich möchte die nächs
4	0	0	
12	0	0	
4	0	0	
0	0	0	
2	0	0	
3	0	0	
0	0	0	
1	0	0	
0	1	0	
1	0	0	
1	0	0	Nein, ich möchte die nächs
5	0	0	1
0	0	0	
3	0	0	
2	0	0	
3	0	0	
3	0	0	
4	0	0	
5	0	0	

July13 Wie viele Prostatas	July13 a) Anzahl Low Risk	July13 b) Anzahl Intermedi	July13 c) Anzahl High Risk
0	0	2	1
4	2	15	12
5	1	3	4
5	0	0	0
5	1	3	3
14	4	5	4
3	1	0	1
4	3	2	1
0	2	4	0
16	1	5	5
2	0	1	1
5	0	1	1
3	0	1	1
3	2	1	1
1	0	0	0
0	0	0	0
5	0	2	0
1	0	0	0
4	0	2	0
5	0	6	1
4	0	0	0
0	0	2	0
5	0	1	1
0	1	1	0
1	0	1	1
5	0	4	2
4	0	1	1

July13 d) Anzahl lokal fortg	July13 Wie viele offene rad	July13 Wie viele roboteras	July13 Wie viele laparosko
0	0	3	0
0	0	29	0
0	8	0	0
0	0	0	0
0	0	7	0
0	1	12	0
0	0	2	0
0	0	6	0
0	0	6	0
0	0	11	0
0	0	2	0
0	1	1	0
0	0	2	0
0	0	4	0
0	0	0	0
0	0	0	0
0	0	0	2
0	0	0	0
0	0	2	0
0	0	7	0
0	0	0	0
0	0	2	0
0	0	2	0
0	0	2	0
0	0	2	0
0	3	3	0
0	0	2	0

July13 Wie viele fokale Th	July13 Ich möchte den Fra	Aug10 Wie viele Prostatas	Aug10 a) Anzahl Low Risk
0		9	0
0		1	6
0	Nein, ich möchte die nächs	9	0
0		8	1
0		3	0
0		13	2
0		3	0
0	Nein, ich möchte die nächs	6	1
0		10	0
0		18	0
0		1	0
0		2	0
0		3	0
0		4	1
0		4	0
0		0	0
0		7	0
0		1	2
0	Nein, ich möchte die nächs	2	0
0	1	7	0
2		0	1
0		1	0
0		6	0
0		6	2
0		5	1
0		4	2
0		4	0

Aug10 b) Anzahl Intermedi	Aug10 c) Anzahl High Risk	Aug10 d) Anzahl lokal fort	Aug10 Wie viele offene rad
2	1	0	0
24	4	0	0
5	4	0	9
4	0	0	0
5	3	0	0
3	8	0	0
1	1	0	0
6	1	0	2
1	1	0	1
7	4	0	0
0	3	0	0
2	1	0	0
3	0	0	0
1	1	0	0
0	0	0	0
1	1	0	0
1	1	0	0
0	0	0	0
1	0	0	1
3	2	0	0
0	0	0	0
0	0	0	0
0	2	0	0
0	0	0	0
1	1	0	0
2	3	0	3
2	3	0	0

Aug10 Wie viele roboteras	Aug10 Wie viele laparosko	Aug10 Wie viele fokale Th	Aug10 Ich möchte den Fra
3	0	0	
34	0	0	
0	0	0	Nein, ich möchte die nächs
5	0	0	
8	0	0	
13	0	0	
2	0	0	
6	0	0	Nein, ich möchte die nächs
1	0	0	
11	0	0	
3	0	0	
3	0	0	
3	0	0	
3	0	0	
0	0	0	
2	0	0	
0	2	0	
2	0	0	
0	0	0	Nein, ich möchte die nächs
5	0	0	1
0	1	1	
0	0	0	
2	0	0	
2	0	0	
3	0	0	
4	0	0	
5	0	0	

Sept07 Wie viele Prostatas	Sept07 a) Anzahl Low Risk	Sept07 b) Anzahl Intermed	Sept07 c) Anzahl High Risl
10	0	2	1
4	5	17	13
7	0	4	1
7	0	3	1
4	0	0	0
14	2	5	3
3	1	0	1
6	0	4	4
0	0	1	3
20	0	5	10
2	0	0	1
0	0	0	0
4	0	0	1
2	1	0	0
1	0	1	0
4	0	1	2
7	0	0	1
4	0	0	0
4	0	1	1
6	1	3	2
3	1	0	0
2	0	0	0
6	0	1	1
6	1	1	1
0	1	1	1
7	2	0	0
5	1	2	2

Sept07 d) Anzahl lokal fort	Sept07 Wie viele offene ra	Sept07 Wie viele roboteras	Sept07 Wie viele laparosko
0	0	3	0
0	0	35	0
0	4	1	0
0	0	4	0
0	0	0	0
0	1	9	0
0	1	1	0
0	1	7	0
0	1	3	0
0	0	15	0
0	0	1	0
0	0	0	0
0	0	1	0
0	0	1	0
0	0	1	0
0	0	3	0
0	1	0	0
0	0	0	0
0	1	1	0
0	0	6	0
0	0	0	1
0	0	0	0
0	0	2	0
0	0	3	0
0	0	3	0
0	0	2	0
0	0	5	0

Sept07 Wie viele fokale Th	Sept07 Ich möchte den Fra	Oct05 Wie viele Prostatast	Oct05 a) Anzahl Low Risk
0		10	0
0		5	10
0	Nein, ich möchte die nächs	7	0
0		8	1
0		3	1
0		12	2
0		3	0
0	Nein, ich möchte die nächs	4	0
0		7	1
0		15	2
0		3	0
0		1	0
0		8	0
0		3	1
0		3	0
0		5	1
0		7	0
0		4	0
0	Nein, ich möchte die nächs	4	0
0	1	5	0
1		2	0
0		1	0
0		4	1
0		9	2
0		3	0
0		10	1
0		6	0

Oct05 b) Anzahl Intermedi	Oct05 c) Anzahl High Risk	Oct05 d) Anzahl lokal fortg	Oct05 Wie viele offene rad
4	1	0	0
14	10	0	0
4	6	0	10
4	2	0	0
3	1	0	0
3	7	0	0
1	1	0	0
4	2	0	0
2	1	0	0
5	5	0	0
1	0	0	0
1	0	0	0
0	1	0	0
2	1	0	0
1	0	0	0
0	1	0	0
1	1	0	0
0	0	0	0
0	0	0	0
1	2	0	0
1	0	0	0
1	0	0	0
1	0	0	0
1	2	0	0
2	2	0	0
2	2	0	3
2	2	0	0

Oct05 Wie viele roboterass	Oct05 Wie viele laparosko	Oct05 Wie viele fokale The	Oct05 Ich möchte den Frag
5	0	0	
34	0	0	
0	0	0	Nein, ich möchte die nächs
7	0	0	
5	0	0	
12	0	0	
2	0	0	
6	0	0	Nein, ich möchte die nächs
4	0	0	
12	0	0	
1	0	0	
1	0	0	
1	0	0	
4	0	0	
1	0	0	
2	0	0	
0	2	0	
0	0	0	
0	0	0	Nein, ich möchte die nächs
3	0	0	1
0	1	1	
1	0	0	
2	0	0	
5	0	0	
4	0	1	
2	0	0	
4	0	0	

Nov02 Wie viele Prostatas	Nov02 a) Anzahl Low Risk	Nov02 b) Anzahl Intermedi	Nov02 c) Anzahl High Risk
6	0	2	1
3	11	21	4
9	1	6	0
7	1	1	1
8	0	2	2
10	2	7	2
4	1	1	1
8	2	3	2
4	0	4	2
20	0	8	7
1	0	0	0
1	0	0	2
7	2	3	0
4	1	1	0
1	0	1	0
2	2	2	0
3	0	2	0
1	1	0	0
3	0	2	0
4	2	2	0
7	0	0	0
0	0	1	0
6	0	2	0
3	1	1	2
2	0	1	0
6	1	4	0
5	0	2	1

Nov02 d) Anzahl lokal fortg	Nov02 Wie viele offene rad	Nov02 Wie viele roboteras	Nov02 Wie viele laparosko
0	0	3	0
0	0	36	0
0	5	2	0
0	0	3	0
0	0	4	0
0	0	11	0
0	0	3	0
0	0	7	0
0	0	6	0
0	0	15	0
0	0	0	0
0	1	1	0
0	0	5	0
0	0	2	0
0	0	1	0
0	0	4	0
0	1	0	1
0	0	1	0
0	0	2	0
0	0	4	0
0	0	0	0
0	0	1	0
0	0	2	0
0	0	4	0
0	0	1	0
0	1	4	0
0	0	3	0

Nov02 Wie viele fokale Th	Nov02 Ich möchte den Fra	Nov30 Wie viele Prostatas	Nov30 a) Anzahl Low Risk
0		10	0
0		9	5
0	Nein, ich möchte die nächs	4	0
0		8	0
0		0	1
0		14	1
0		3	1
0	Nein, ich möchte die nächs	3	1
0		4	0
0		25	0
0		1	0
0		1	0
0		10	0
0		3	1
0		1	1
0		5	0
0		8	0
0		3	0
0	Nein, ich möchte die nächs	4	0
0	1	7	2
1		5	0
0		0	0
2		3	0
0		6	1
0		2	1
0		9	1
0		5	0

Nov30 b) Anzahl Intermedi	Nov30 c) Anzahl High Risk	Nov30 d) Anzahl lokal fortg	Nov30 Wie viele offene rad
2	0	0	0
18	7	0	0
4	4	0	7
3	1	0	0
4	1	0	0
7	5	0	1
1	1	0	0
1	2	0	1
5	1	0	0
7	7	0	0
0	2	0	0
2	0	0	1
1	1	0	0
3	1	0	0
0	0	0	1
1	1	0	0
0	0	0	0
1	0	0	0
1	1	0	1
3	1	0	0
0	0	0	0
0	0	0	0
1	0	0	0
2	0	0	0
1	1	0	0
4	0	0	4
0	3	0	0

Nov30 Wie viele roboteras	Nov30 Wie viele laparosko	Nov30 Wie viele fokale Th	Nov30 Ich möchte den Fra
2	0	0	
30	0	0	
1	0	0	Nein, ich möchte die nächs
4	0	0	
6	0	0	
12	0	0	
3	0	0	
3	0	0	Nein, ich möchte die nächs
6	0	0	
14	0	0	
2	0	0	
1	0	0	
2	0	0	
5	0	0	
0	0	0	
2	0	0	
0	0	0	
1	0	0	
1	0	0	Nein, ich möchte die nächs
6	0	1	1
0	0	0	
0	0	0	
1	0	1	
3	0	0	
3	0	0	
1	0	0	
3	0	0	

Dec14 Wie viele Prostatas	Dec14 a) Anzahl Low Risk	Dec14 b) Anzahl Intermedi	Dec14 c) Anzahl High Risk
10	0	0	0
7	5	16	7
3	0	5	4
7	0	2	1
6	1	2	1
11	1	3	4
4	2	0	0
10	1	1	4
10	0	1	2
15	0	10	5
3	3	1	0
1	0	0	1
5	1	1	1
2	1	1	0
0	0	1	0
3	3	0	0
9	0	0	0
1	2	1	0
3	0	0	0
9	2	3	1
4	0	0	2
1	0	2	0
4	0	2	0
5	0	0	1
6	0	0	0
10	0	4	1
4	0	2	2

Dec14 d) Anzahl lokal fortg	Dec14 Wie viele offene rad	Dec14 Wie viele roboteras	Dec14 Wie viele laparosko
0	0	0	0
0	0	28	0
0	8	1	0
0	0	3	0
0	0	4	0
0	1	7	0
0	0	2	0
0	0	6	0
0	1	2	0
0	0	15	0
0	0	4	0
0	0	1	0
0	0	3	0
0	0	2	0
0	0	1	0
0	0	3	0
0	0	0	0
0	0	3	0
0	0	0	0
0	0	6	0
0	0	0	2
0	0	2	0
0	0	2	0
0	0	1	0
0	0	0	0
0	1	4	0
0	0	4	0

Dec14 Wie viele fokale Th	Dec14 Ich möchte den Fra	Jan11 Wie viele Prostatast	Jan11 a) Anzahl Low Risk
0		12	1
0		11	2
0	Nein, ich möchte die nächs	7	0
0		9	0
0		6	0
0		15	6
0		2	0
0	Nein, ich möchte die nächs	2	1
0		7	0
0		25	0
0		2	1
0		3	0
0		7	1
0		3	1
0		0	0
0		4	0
0		8	0
0		1	2
0	Nein, ich möchte die nächs	2	0
0	1	9	1
0		3	0
0		1	0
2		3	0
0		5	0
0		4	1
0		12	1
0		5	1

Jan11 b) Anzahl Intermedi	Jan11 c) Anzahl High Risk	Jan11 d) Anzahl lokal fortg	Jan11 Wie viele offene rad
2	1	0	0
21	12	0	0
7	4	0	10
6	2	0	0
2	1	0	0
11	4	0	18
1	1	0	0
4	1	0	1
2	1	0	0
9	4	0	0
0	2	0	0
0	1	0	0
4	1	0	0
0	0	0	0
1	0	0	0
2	2	0	0
0	1	0	0
0	1	0	0
0	0	0	0
6	1	0	0
0	0	0	0
2	0	0	0
2	0	0	0
4	1	0	0
1	2	0	0
5	2	0	2
1	2	0	0

Jan11 Wie viele roboterass	Jan11 Wie viele laparosko	Jan11 Wie viele fokale The	Jan11 Ich möchte den Frag
4	0	0	
35	0	0	
1	0	0	Nein, ich möchte die nächs
8	0	0	
3	0	0	
3	0	0	
2	0	0	
5	0	0	Nein, ich möchte die nächs
3	0	0	
13	0	0	
3	0	0	
1	0	0	
6	0	0	
1	0	0	
1	0	0	
4	0	0	
0	1	0	
3	0	0	
0	0	0	Nein, ich möchte die nächs
8	0	0	1
0	0	0	
2	0	0	
2	0	1	
5	0	0	
4	0	0	
6	0	1	
4	0	0	

Feb08 Wie viele Prostatas	Feb08 a) Anzahl Low Risk	Feb08 b) Anzahl Intermedi	Feb08 c) Anzahl High Risk
12	0	0	4
8	5	20	9
9	0	8	2
8	0	4	2
4	0	4	1
10	2	6	1
2	0	2	0
7	1	4	1
6	1	3	1
25	0	4	11
3	2	1	0
1	0	1	0
5	0	2	1
4	0	2	2
1	1	0	0
4	0	1	1
7	0	0	0
1	1	0	0
1	0	0	0
8	1	1	2
1	0	1	0
1	0	2	1
4	0	1	1
5	0	3	0
5	1	2	0
11	0	2	3
4	0	1	2

Feb08 d) Anzahl lokal fortg	Feb08 Wie viele offene rad	Feb08 Wie viele roboteras	Feb08 Wie viele laparosko
0	0	4	0
0	0	34	0
0	10	0	0
0	0	6	0
0	0	5	0
0	0	9	0
0	0	2	0
0	0	6	0
0	0	5	0
0	0	15	0
0	0	3	0
0	0	1	0
0	0	0	3
0	0	4	0
0	0	1	0
0	0	2	0
0	0	0	0
0	0	1	0
0	0	0	0
0	0	4	0
0	0	0	1
0	0	3	0
0	0	2	0
0	0	3	0
0	0	3	0
0	0	5	0
0	0	3	0

Feb08 Wie viele fokale Th	BL_1 Pat	BL_1 RAD	BL_2 Pat
0	2	2	2
0	33	33	29
0	15	15	11
0	7	7	7
0	8	8	7
0	18	18	13
0	3	3	3
0	4	4	8
0	4	4	5
0	12	12	15
0	5	5	4
0	4	4	2
0	6	6	5
0	2	2	5
0	2	2	2
0	4	4	4
0	3	3	3
0	0	0	1
0	0	0	1
0	5	5	9
2	1	1	2
0	2	2	2
0	3	3	3
0	6	6	4
0	2	2	3
0	8	8	8
0	6	6	5

BL_2 RAD	March16_Pat	March16_RAD	April20_Pat
2	2	2	4
29	29	29	31
11	7	7	12
7	7	7	5
7	7	7	7
13	9	9	12
3	2	2	2
8	8	8	2
5	7	7	6
15	14	14	13
4	3	3	4
2	0	0	3
5	3	3	5
5	5	5	6
2	0	0	2
4	4	4	5
3	2	2	0
1	0	0	0
1	0	0	0
9	8	8	6
2	1	1	0
2	2	2	5
3	2	2	3
4	3	3	1
3	7	7	5
8	9	9	4
5	7	7	5

April20_RAD	May18_PAT	May18_RAD	June15_PAT
4	2	2	2
31	27	27	34
12	7	7	11
5	3	3	6
7	3	3	0
12	8	8	13
2	2	2	2
2	2	2	7
6	4	4	4
13	18	18	12
4	1	1	4
3	0	0	0
5	3	3	2
6	0	0	3
2	0	0	1
5	2	2	1
0	0	0	1
0	2	2	1
0	0	0	2
6	7	7	5
0	1	1	0
5	0	0	3
3	0	0	2
1	1	1	3
5	3	3	3
4	6	6	8
5	2	2	5

June15_RAD	July13_PAT	July13_RAD	Aug10_PAT
2	3	3	3
34	29	29	34
11	8	8	9
6	0	0	5
0	7	7	8
13	13	13	13
2	2	2	2
7	6	6	8
4	6	6	2
12	11	11	11
4	2	2	3
0	2	2	3
2	2	2	3
3	4	4	3
1	0	0	0
1	0	0	2
1	2	2	2
1	0	0	2
2	2	2	1
5	7	7	5
0	0	0	1
3	2	2	0
2	2	2	2
3	2	2	2
3	2	2	3
8	6	6	7
5	2	2	5

Aug10_RAD	Sept07_PAT	Sept07_RAD	Oct05_PAT
3	3	3	5
34	35	35	34
9	5	5	10
5	4	4	7
8	0	0	5
13	10	10	12
2	2	2	2
8	8	8	6
2	4	4	4
11	15	15	12
3	1	1	1
3	0	0	1
3	1	1	1
3	1	1	4
0	1	1	1
2	3	3	2
2	1	1	2
2	0	0	0
1	2	2	0
5	6	6	3
1	1	1	1
0	0	0	1
2	2	2	2
2	3	3	5
3	3	3	4
7	2	2	5
5	5	5	4

Oct05_RAD	Nov02_PAT	Nov02_RAD	Nov30_PAT
5	3	3	2
34	36	36	30
10	7	7	8
7	3	3	4
5	4	4	6
12	11	11	13
2	3	3	3
6	7	7	4
4	6	6	6
12	15	15	14
1	0	0	2
1	2	2	2
1	5	5	2
4	2	2	5
1	1	1	1
2	4	4	2
2	2	2	0
0	1	1	1
0	2	2	2
3	4	4	6
1	0	0	0
1	1	1	0
2	2	2	1
5	4	4	3
4	1	1	3
5	5	5	5
4	3	3	3

Nov30_RAD	Dec14_PAT	Dec14_RAD	Jan11_PAT
2	0	0	4
30	28	28	35
8	9	9	11
4	3	3	8
6	4	4	3
13	8	8	21
3	2	2	2
4	6	6	6
6	3	3	3
14	15	15	13
2	4	4	3
2	1	1	1
2	3	3	6
5	2	2	1
1	1	1	1
2	3	3	4
0	0	0	1
1	3	3	3
2	0	0	0
6	6	6	8
0	2	2	0
0	2	2	2
1	2	2	2
3	1	1	5
3	0	0	4
5	5	5	8
3	4	4	4

Jan11_RAD	Feb08_PAT	Feb08_RAD
4	4	4
35	34	34
11	10	10
8	6	6
3	5	5
21	9	9
2	2	2
6	6	6
3	5	5
13	15	15
3	3	3
1	1	1
6	3	3
1	4	4
1	1	1
4	2	2
1	0	0
3	1	1
0	0	0
8	4	4
0	1	1
2	3	3
2	2	2
5	3	3
4	3	3
8	5	5
4	3	3
